# Increased predation of nutrient-enriched aposematic prey

**DOI:** 10.1098/rspb.2013.3255

**Published:** 2014-04-22

**Authors:** Christina G. Halpin, John Skelhorn, Candy Rowe

**Affiliations:** Centre for Behaviour and Evolution, Newcastle University, Newcastle upon Tyne, Tyne and Wear, UK

**Keywords:** educated predator, prey defences, nutrients, toxic prey, mimicry

## Abstract

Avian predators readily learn to associate the warning coloration of aposematic prey with the toxic effects of ingesting them, but they do not necessarily exclude aposematic prey from their diets. By eating aposematic prey ‘educated’ predators are thought to be trading-off the benefits of gaining nutrients with the costs of eating toxins. However, while we know that the toxin content of aposematic prey affects the foraging decisions made by avian predators, the extent to which the nutritional content of toxic prey affects predators' decisions to eat them remains to be tested. Here, we show that European starlings (*Sturnus vulgaris*) increase their intake of a toxic prey type when the nutritional content is artificially increased, and decrease their intake when nutritional enrichment is ceased. This clearly demonstrates that birds can detect the nutritional content of toxic prey by post-ingestive feedback, and use this information in their foraging decisions, raising new perspectives on the evolution of prey defences. Nutritional differences between individuals could result in equally toxic prey being unequally predated, and might explain why some species undergo ontogenetic shifts in defence strategies. Furthermore, the nutritional value of prey will likely have a significant impact on the evolutionary dynamics of mimicry systems.

## 1. Introduction

Many insects use chemical defences in an attempt to ward off predators. These defences are often combined with conspicuous warning colours and/or markings, a mode of defence known as aposematism [1–4]. To date, empirical and theoretical work surrounding the evolution of aposematism and mimicry (where sympatric species share the same warning pattern) has often focused on understanding how defences promote avoidance in predators [2–6]. Yet, it is clear that predators do not simply avoid aposematic prey, but continue to include them in their diets even when they know that they contain toxins [7–11]. This is because although they are toxic, aposematic prey also contain valuable nutrients [12–14], and ‘educated’ predators that have learned about the prey in their environment should trade-off the cost of ingesting a toxin with the benefit of gaining nutrients when deciding whether or not to eat toxic prey [15–17]. However, while we know that educated predators use toxin content in their predatory decisions, we do not know whether these decisions are affected by preys' nutritional content.

Chemically defended insects will naturally vary in their toxicity and their nutritional value, both within and between species [8,18,19]. Prey toxicity is known to be important in foraging decisions, as educated predators use what they have learned about prey toxin content to select those prey known to contain less toxin [20–22]. This becomes increasingly important as a predator's toxin burden increases and it needs to avoid consuming too much toxin [14,23]. The nutritional state of predators also affects their foraging decisions: predators that are in a poor energetic state increase their intake of toxic prey in order to gain more nutrients [10,13,22,24–26]. Consequently, we would expect that the nutritional content of aposematic prey would also influence predators' decisions to eat them [15,16,27,28]. It is therefore crucial that we understand how predators use information about the nutrients that the prey contain as this is likely to have a significant impact on our understanding of the evolution of prey defence strategies.

Here, we provide the first direct test of how birds evaluate toxic prey based on their nutritional content. We use an established system of wild-caught European starlings (*Sturnus vulgaris*) foraging on live undefended and defended mealworms (*Tenebrio molitor*) [7,14,29], where the nutritional content of the defended prey can be experimentally manipulated. Our findings have important implications for our current understanding of how avian predators impact on the life-history strategies of insects and the evolution of insect defences.

## 2. Material and methods

### (a) Subjects and housing

Eight (four male and four female) wild-caught European starlings (*Sturnus vulgaris*) were caught under licence (English Nature 20093299) and kept in indoor free-flight aviaries. During experimental testing, subjects were housed in pairs in cages measuring 150 × 45 × 45 cm. Each cage had a removable, opaque divider that divided the cage in half. This was used to separate each pair of birds prior to training and experimental sessions. On each side of the cage, there was a drawer measuring 45 × 75 cm, with a spring-loaded flap facing the front through which prey could be presented. Water was available at all times and food (chick crumbs, fresh fruit and Orlux insect patee) was available ad libitum, except when birds were food deprived for 1.5 h before a session (see below). After the experiment, the birds were returned to free-flight aviaries before being released at the same site from which they were caught.

### (b) Prey manipulations

We used mealworms (*Tenebrio molitor*) of similar weight (0.19–0.21 g) to prey. Three prey types were created for use in the experimental sessions by injecting the mealworms with different solutions; undefended prey were injected with 0.04 ml water, low-nutrient-defended prey (LN prey) were injected with 0.02 ml of a 4% quinine solution (4 g quinine sulfate, Sigma Aldrich, in 100 ml water) and 0.02 ml of water, and high-nutrient-defended prey (HN prey) were injected with 0.02 ml of a 4% quinine solution and 0.02 ml of a dietary supplement solution (15 g of ProBoost SuperMax powder in 100 ml of water). The dietary supplement consisted of 84% protein, 1% fat, 5% ash, flourish, vitamins and minerals. We chose this dietary manipulation because starlings are known to prefer foods that contain more protein [30]. The mealworms were all injected through the mouthparts using a hypodermic needle. Quinine has been used widely as an aversant in learning experiments [5,31–33] and previous work has shown that it cannot be tasted when injected into mealworms in this manner [23,29]. In addition, we know from previous experiments using the same amounts of quinine [34,35] that once the starlings have reached a daily asymptotic attack rate on the quinine-injected mealworms, this remains stable, thus demonstrating that there is not any accumulation of quinine in the birds' systems across days.

### (c) Training sessions

A white curtain erected in front of the cage visually isolated birds during training and experimental sessions. Birds were observed via video cameras linked to television monitors, and sessions were recorded for further analysis. Birds were initially trained to eat unmanipulated mealworms (i.e. not injected with any solution) out of Petri dishes on a white background. They were given a single training session on each of two consecutive days, which consisted of a sequence of 24 singly presented mealworms. A presentation was made every 3 min, and birds were given 1 min to attack a mealworm, after which time the Petri dish was removed. After 2 days, all birds ate all the mealworms presented to them, confirming that satiation would not be a limiting factor to the number of prey eaten in the experimental sessions. Once they met this criterion, we moved on to the experimental sessions.

### (d) Experimental sessions

From day 3, birds were given one experimental session per day for 15 consecutive days. In these sessions, each bird was given a sequence of 12 undefended and 12 defended mealworms presented singly in Petri dishes. Undefended and defended prey were given distinct colour signals in the form of green and purple coloured paper discs that were placed in the Petri dishes underneath the mealworm. Colours were counter-balanced across birds to control for any potential colour biases [32]. The nutritional content of the defended prey was either high or low depending on the session number. All birds were initially given HN-defended prey (sessions 1–5) followed by LN-defended prey (sessions 6–10). Then, to ensure that any observed effect was not simply an order effect, birds were again given HN-defended prey (sessions 11–15). The numbers of undefended and defended prey eaten were recorded each day to determine when a stable asymptotic attack rate had been reached on the defended prey type. This was to ensure that the birds were knowledgeable about the prey and were making informed foraging decisions before the nutritional content of the defended prey was changed. The order in which undefended and defended prey were presented to a bird within a session was randomized for all except the first two sessions in each phase of nutrient manipulation (i.e. sessions 1–2, 6–7 and 11–12), when undefended and defended prey were presented in ‘blocks’ of four. This was to facilitate learning (K Ashbrook, J Skelhorn and C Rowe 2007, unpublished data).

### (e) Data analysis

We know that starlings will not completely avoid quinine-injected mealworms but that they reach a stable asymptotic attack rate once they have learned about the quinine content of the prey and become ‘educated’ [14]. As we were interested in comparing the foraging behaviour of educated predators, we needed to establish that they had reached stable asymptotic attack rates on defended prey when these were protein-injected and when they were not. Comparing the asymptotes for HN- and LN-defended prey allowed us to test for differences in the mortality of these prey based on nutritional differences (raw data for each bird supplied as the electronic supplementary material). Owing to the relatively small sample size we used non-parametric tests. To determine when birds had reached asymptote, we ran a series of Friedman tests on the attack data, with prey type and session number as repeated measures. For each of the three sets of sessions, where protein content changed from high to low and back to high, we initially included all five sessions (1–5, 6–10 and 11–15), then the last four sessions (2–5, 7–10, 12–15), then the last three sessions (3–5, 8–10, 13–15), until there was no significant effect of session number [34,35]. Once we had established the three asymptotic attack rates for the defended prey, we carried out Wilcoxon signed-rank tests to test our prediction that the asymptotes would decrease when we switched from HN- to LN-defended prey, and increase once again when we replaced LN- with HN-defended prey.

## 3. Results

**Figure 1.**
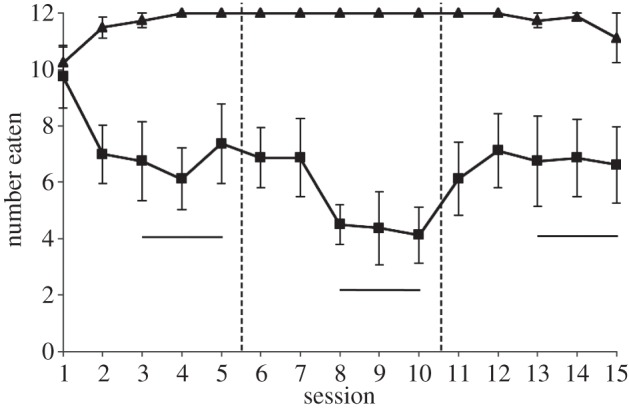
The mean numbers (±s.e.) of undefended (triangles) and defended (squares) prey eaten across sessions. The dashed lines mark when the protein content of defended prey was changed; starting with HN prey (sessions 1–5), followed by LN prey (sessions 6–10) and finally HN prey (sessions 11–15). The horizontal lines denote sessions where there are no significant differences in the number of defended prey eaten.

We initially analysed the data across sessions to establish that the birds had learned to discriminate between undefended and defended prey. Although birds ate the same number of undefended and defended prey in session 1 (Z = 0.00, *p* = 1.00; figure 1), by session 2 they were already eating more undefended than defended prey in a session (*Z* = −2.384, *p* = 0.017; figure 1). Indeed, from session 3 onwards, birds predominantly ate all of the undefended prey (figure 1). However, the number of defended prey eaten varied across the 15 sessions in line with changes in their nutrient content (figure 1). When the nutritional content of the defended prey was either increased or decreased, i.e. within each of the three 5-session periods, the birds reached asymptotic attack rates by the third session. In other words, there was no significant difference in the numbers of defended prey eaten across sessions 3–5 (HN-defended prey: 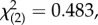 *p* = 0.786), sessions 8–10 (LN-defended prey: 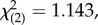 *p* = 0.565) or sessions 13–15 (HN-defended prey: 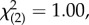 *p* = 0.607) (figure 1). Therefore, we used the data from these sessions to compare the asymptotic ingestion of defended prey when their nutritional content differed (see the electronic supplementary material for raw data).

**Figure 2.**
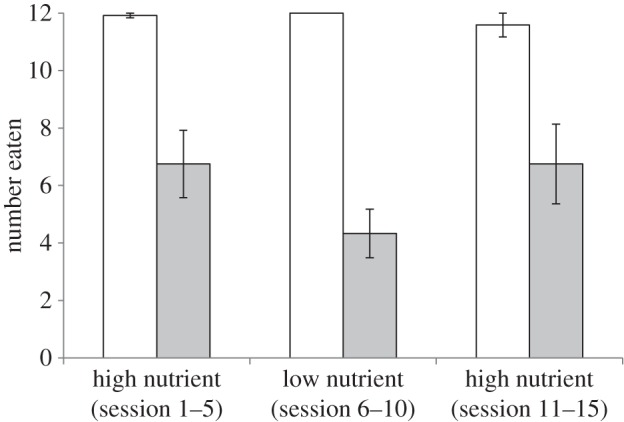
The mean number (±s.e.) of undefended prey (white bars) and defended prey (grey bars) eaten at asymptote at the start (HN (1)), middle (LN) and end (HN (2)) of the experiment.

As predicted, the asymptotic attack rate on LN-defended prey was significantly lower than that on HN-defended prey, both at the beginning and at the end of the experiment (*Z* = −2.316, *p* = 0.021 and *Z* = −2.111, *p* = 0.035, respectively; figure 2), indicating that birds were willing to consume more of the defended prey when the nutritional benefit of eating them was higher. Notably, there was no difference in the numbers of HN-defended prey eaten across sessions 3–5 and sessions 13–15 (*Z* = −0.140, *p* = 0.888; figure 2). This shows that the birds were changing their foraging decisions according to the nutritional content of the defended prey.

## 4. Discussion

We have clearly demonstrated that avian predators are prepared to eat more chemically defended prey when they are nutritionally enriched compared with when they are not. Since we have previously shown that increasing the nutrition available from alternative undefended prey *decreases* the ingestion of toxic prey [35], which is consistent with findings from other studies [27,28,36,37], we can be sure that predators' increased willingness to consume nutritionally enriched defended prey is owing to the increase in the nutrient content of defended prey rather than the increase in overall nutrient availability. This finding has significant implications for our understanding of how predators assess prey profitability, and consequently the evolution of aposematism and mimicry, and the life-history strategies of defended prey.

Although it is generally assumed that prey containing the same amount of toxin are equally unprofitable [32,38,39], and that profitability is negatively correlated with toxicity [40,41], our data provide empirical support for a recent theoretical prediction that neither of these assumptions need necessarily be correct [27]. Predators' willingness to eat aposematic prey is affected by their nutrient content, meaning that equally toxic prey that differ in their nutrient content will also differ in their profitability to predators; and that prey with higher toxin concentrations may be less defended than those with lower toxin concentrations if they also contain more nutrients. In short, toxicity alone is not a reliable measure of prey profitability: we need to consider both the toxicity and the nutritional value of aposematic prey in order to understand just how unprofitable aposematic prey are to their predators.

This view of prey profitability changes the way we view the evolution of defensive strategies, such as toxicity, aposematism and mimicry. At a very basic level, the nutritional value of prey will influence the degree to which they have to invest in costly toxins in order to gain protection from predators: with more nutritious prey having to invest more in order to gain the same level of protection. However, there are also more complex ways in which our view of prey profitability changes the way we think about the evolution of prey defences. One of the biggest questions in the study of Müllerian mimicry is whether a less toxic mimic dilutes the defence of a more toxic mimic (i.e. it is a quasi-Batesian mimic), or whether both co-mimics mutually benefit from sharing the same warning pattern (i.e. they are true Müllerian mimics) [40–42]. However, we would argue that this is an over-simplified view. Our results support the prediction of Turner & Speed [43] that even equally toxic Müllerian mimics may be unequally defended if one is more nutritious than the other [43], and lead to the counterintuitive prediction that the relationship between equally toxic Müllerian mimics could under some circumstances be parasitic and quasi-Batesian.

We may also need to re-think our understanding of the evolutionary dynamics of Batesian mimicry, where a palatable prey species dilutes the defence of a toxic model. In line with a recent prediction that body size impacts on mimicry dynamics [44], our data suggest that the degree to which a Batesian mimic degrades the protection of its model may in fact depend on its nutritional content. Furthermore, together with the idea that low nutrient content in prey could be a form of defence [45,46], our findings provide empirical support for the theory that a nutrient-poor Batesian mimic may not be parasitic on its model at all [43], and could even enhance the protection of a toxic model if it reduces attack rates on the model–mimic complex. This view of prey profitability clearly suggests that the evolutionary dynamics of mimicry are considerably more complex than previously thought. While some mathematical models have considered how the ‘profitability’ of mimics, in terms of abundance or handling times, impacts on predator foraging strategies [27,47], none has yet included variability in the nutrient content of the individual prey types themselves. This makes it impossible to know how nutrients and toxins co-evolve in prey. Mathematical models of the selection pressures generated by predators that base their foraging decisions on the nutritional content of prey as well as their toxin content may well reveal further insights into the evolution of prey defences (see also [44]).

Our data also suggest that predation may have a significant impact on the life-history strategies of aposematic prey. As predation rates on aposematic prey increase with increasing nutritional value, the benefits of aposematism are likely to change over ontogeny as prey grow and become more nutritious, and could even decrease if toxicity remains constant. For example, selection may favour individuals that increase their investment in costly chemical defences as they grow, which could explain the correlation between body size and the total toxin content found in some aposematic species [48]. However, there may come a point where the cost of investing more heavily in defence chemicals outweighs the benefits of increasing in size (e.g. increased fecundity) and selection may favour a reduction in adult size. Alternatively, selection may favour individuals that switch between alternative defensive strategies as the relative costs and benefits of these strategies changes over ontogeny. Such ontogenetic shifts in defensive strategies are common [49,50], but are generally explained by assuming either that the benefit of crypsis changes with size [51], or that larger prey have had more time/opportunity to synthesize/sequester defensive compounds [49,50], rather than assuming that the benefit of aposematism decreases with increasing size. Our results therefore suggest that nutritional value, toxin content and visual signals of prey are likely to co-evolve in response to predation by educated predators, and challenge us to take a broader life-history approach to understand the fitness consequences of aposematism and mimicry, rather than one focused on predator aversion learning.

Notably, while we have discussed our results in terms of the overall nutritional content of prey, predators' decisions to eat toxic prey are likely to be driven by the specific micronutrients and/or macronutrients that prey contain [16,52–54]. Consequently, predators' decisions to eat toxic prey in the wild could be influenced by a range of biotic and abiotic variables across trophic levels. This is because a predator's need for particular nutrients will depend on the availability of those nutrients from prey in the environment. This, in turn, will be influenced by the quality and quantity of available resources for the prey (particularly from their host plants), which will be affected by a number of factors, including inter- and intraspecific competition, soil nutrient levels, weather and how plants respond to their environment. Furthermore, prey could be under selection to change their own diets in a way that decreases their intake of the nutrients that are essential to their predators. Clearly, this strategy could only evolve if it provided a selective advantage to an initial rare mutant [55], for example, when predators detect the poor nutritional quality of prey upon attack and either release it unharmed, eat only part of the organism, or subsequently reduce their attacks on nearby kin. It may thus be more likely to occur in prey that advertise their nutritional value, those that can survive being partly consumed or those that live in kin groups [55–57]. Although it remains to be tested, it is thought that some organisms use reduced nutritional quality as a form of defence [45,46], and it is possible that the extent to which this occurs in nature could be driven by the specific nutritional requirements of predators. Overall, it is clear that the nutritional content of prey will be influenced by a complex interaction of top-down and bottom-up factors, which will have knock on effects for predators' foraging decisions. Furthermore, understanding these complex multi-trophic interactions will be a challenging but necessary step in understanding the evolution of defensive strategies in prey.

In conclusion, we have provided the first empirical demonstration that predators' perceptions of the profitability of defended prey are influenced by the prey's nutritional value. In addition, we have outlined some of the ways in which the nutritional content of defended prey could influence the evolution of both prey's defensive and life-history strategies. Furthermore, we are confident that (i) incorporating the nutritional content of prey as a variable in mathematical models of the evolution of aposematism and mimicry, (ii) empirically testing the hypotheses on mimicry dynamics that we present above and (iii) investigating the cognitive mechanisms that predators use to trade-off the toxic costs and nutritional benefits of consuming defended prey will prove fruitful areas for future research, and lead to further significant insights into the evolution of prey defences.

## Notes

The experiment was conducted under Local Ethical Committee approval (ERC Project ID: 266), and in accordance with ASAB's Guidelines for the Treatment of Animals in Behavioural Research and Teaching.
